# Procalcitonin in acute and chronic coronary syndromes: Diagnostic biomarker of coronary inflammation

**DOI:** 10.17305/bb.2025.12915

**Published:** 2025-09-16

**Authors:** Ozan Sakarya, Burak Toprak, Rıdvan Bora

**Affiliations:** 1 Department of Cardiology, Turgutlu State Hospital, Manisa, Türkiye; 2 Department of Cardiovascular Surgery, Mersin City Education and Research Hospital, Mersin, Türkiye; 3 Department of Cardiology, Tarsus State Hospital, Mersin, Türkiye

**Keywords:** Procalcitonin, coronary artery disease, acute coronary syndrome, chronic coronary syndrome, inflammation

## Abstract

Procalcitonin (PCT) is classically a biomarker of bacterial infection, but its role in cardiovascular inflammation—particularly in coronary artery disease (CAD)—is less well defined. Evidence linking PCT with disease extent and outcomes across acute coronary syndrome (ACS) and chronic coronary syndrome (CCS) remains limited. We compared PCT levels among ACS, CCS, and angiographic controls; examined associations with inflammatory burden and anatomic complexity (SYNTAX score); and evaluated diagnostic performance and short- and intermediate-term prognostic value. In this single-center retrospective study, 477 consecutive adults undergoing diagnostic coronary angiography (December 2019–March 2020) were categorized as ACS (*n* ═ 190), CCS (*n* ═ 202), or controls with normal epicardial arteries (*n* ═ 85). Demographic, laboratory, and angiographic data were collected. PCT was measured within 24 h of admission. Multivariable logistic regression (using log_10_-transformed PCT) assessed independent associations with ACS and CCS. Correlations tested relationships with SYNTAX, C-reactive protein (CRP), and troponin-I. Receiver operating characteristic (ROC) analyses quantified discrimination. In ACS, outcomes were compared by PCT ≥ 0.25 ng/mL. Median PCT was higher in ACS and CCS than in controls (both *P* < 0.001). Log_10_-PCT independently predicted disease presence in ACS (OR 4.30, 95% CI 2.00–9.20, *P* < 0.001) and CCS (OR 2.81, 95% CI 1.43–5.54, *P* ═ 0.003). In CCS, PCT correlated weakly but significantly with SYNTAX score (*r* ═ 0.274, *P* ═ 0.002); no meaningful correlations with SYNTAX, CRP, or troponin-I were observed in ACS. PCT showed moderate diagnostic accuracy (AUC 0.791 for ACS; optimal cut-off 0.25 ng/mL, sensitivity 82.4%, specificity 65.3%; and AUC 0.763 for CCS; optimal cut-off 0.30 ng/mL, sensitivity 89.4%, specificity 54.0%; all *P* < 0.001). In ACS, PCT ≥ 0.25 ng/mL was not associated with higher in-hospital mortality, 1-year all-cause mortality, or major adverse cardiovascular events. PCT reflects inflammatory burden and the presence of CAD in both ACS and CCS and remains an independent predictor of disease presence, but its prognostic utility—particularly in ACS—is limited. PCT should complement, not replace, established biomarkers and anatomical scoring systems in clinical decision-making. Prospective, multicenter studies with serial PCT measurements are warranted to refine its clinical role.

## Introduction

Cardiovascular diseases (CVDs) remain a leading cause of mortality and morbidity globally. Representing nearly half of all non-communicable diseases, CVD poses a significant public health challenge, with projections indicating over 17 million deaths annually by 2030 [1]. Among the various CVDs, coronary artery disease (CAD) is particularly critical due to its high prevalence and its link to sudden cardiac events. CAD presents in two primary clinical forms: acute coronary syndrome (ACS) and chronic coronary syndrome (CCS).

According to the 2023 and 2024 guidelines of the European Society of Cardiology, ACS encompasses clinical presentations characterized by acute chest pain, dynamic electrocardiographic changes—such as ST-segment elevation or depression—and elevated cardiac biomarkers, notably high-sensitivity troponin. This category includes ST-elevation myocardial infarction, non-ST-elevation myocardial infarction, and unstable angina. Conversely, CCS is characterized by stable patterns of exertional or predictable chest pain without acute changes in electrocardiographic findings or biomarker levels, typically diagnosed through clinical assessment and non-invasive or invasive imaging tests [2, 3]. Despite their differing clinical trajectories, both ACS and CCS share a common underlying pathophysiological process: atherosclerosis [4].

Atherosclerosis is a chronic, progressive vascular disease characterized by lipid accumulation, endothelial dysfunction, immune system activation, and inflammatory responses. This condition leads to luminal narrowing of the arteries and increases the risk of plaque instability and sudden thrombotic events. Consequently, recent research has emphasized that atherosclerosis should be viewed not only as a lipid-driven pathology but also as an inflammatory disease [5].

In clinical practice, various biomarkers are utilized to assess inflammation. While markers such as C-reactive protein (CRP) and interleukin-6 (IL-6) have been extensively studied in relation to the atherosclerotic process, procalcitonin (PCT), a known marker of infection, has recently garnered attention in this context [6, 7]. Under normal conditions, PCT is an inactive propeptide secreted by thyroid C cells and subsequently converted into calcitonin. However, during systemic inflammation—particularly in response to bacterial endotoxins and proinflammatory cytokines (IL-1β, TNF-α, IL-6)—PCT is actively synthesized by various parenchymal organs, including the liver, lungs, and intestines, as well as by the monocyte–macrophage system, leading to significantly elevated serum levels [8].

The direct correlation between PCT and systemic inflammatory responses positions PCT as a potential biomarker not only for infections but also for various inflammatory conditions. Recent studies indicate that PCT levels can elevate in non-infectious scenarios, particularly during cardiovascular events. Consequently, PCT has been proposed as a marker of the inflammatory response, offering prognostic insights in conditions such as ACS, heart failure, and certain stable coronary syndromes [9, 10]. This has prompted the consideration of PCT as a “non-specific inflammatory biomarker” within the context of atherosclerotic diseases.

Nevertheless, investigations into the relationship between PCT and different CAD subtypes, such as ACS and CCS remain limited and have produced conflicting findings. Additionally, the correlation between PCT levels and the angiographic extent or severity of CAD has yet to be definitively established. The SYNTAX (Synergy Between Percutaneous Coronary Intervention with TAXUS and Cardiac Surgery) score, developed to assess the anatomical complexity and extent of CAD lesions, serves as a crucial reference in this study [11].

This study aims to compare serum PCT levels among patients with ACS, CCS, and controls with angiographically normal epicardial coronary arteries. If significant differences are identified, the research will further explore the associations of these differences with disease extent and severity, as well as their prognostic implications within the ACS cohort. The ultimate objective is to provide new and clearer insights into the role of PCT in the atherosclerotic process.

## Materials and methods

### Data collection

#### Study design

This study presents a retrospective observational clinical investigation conducted within the Department of Cardiology at the Faculty of Medicine, Mersin University. We reviewed the medical records and digital archives of patients who presented to the cardiology department with a preliminary diagnosis of CAD and underwent diagnostic coronary angiography between December 1, 2019, and March 15, 2020. Although the study period coincided with the early phase of the COVID-19 pandemic, all data were collected prior to widespread national lockdowns and significant reorganization of catheterization laboratory schedules.

The investigation was conducted in a tertiary referral center situated in a metropolitan area with a population exceeding three million, serving as a regional hub for cardiovascular diagnostics and interventions. During the study period (December 1, 2019 to March 15, 2020; 106 days), a total of 510 diagnostic coronary angiograms were performed, averaging approximately 4.8 procedures per day. Of these, 33 cases were excluded due to incomplete data, resulting in a final analytical cohort of 477 patients. Only those patients with complete clinical, laboratory, and imaging data at the time of admission were included. Patients lacking essential clinical, laboratory, or imaging data were excluded from the analysis to minimize bias associated with incomplete datasets.

Among the 510 initially screened patients, 33 were excluded: 14 had incomplete laboratory results, 11 were missing imaging data, and 8 had incomplete clinical records. The final cohort included 477 patients (190 with ACS, 202 with CCS, and 85 controls). Baseline characteristics of the excluded patients did not significantly differ in terms of age, sex distribution, or major cardiovascular risk factors compared to the included cohort, thereby minimizing the likelihood of systematic bias.

The dataset encompassed demographic information, medical history, laboratory results, and coronary angiography images collected at admission. No interventions were made regarding patient management during the study period; data analysis relied solely on the evaluation of existing records. To mitigate potential selection bias inherent to the retrospective nature of the study, predetermined and standardized inclusion and exclusion criteria were applied. All data were retrieved from the hospital information management system and archived digital angiography images, with the integrity of the records verified by two independent investigators.

#### Data collection

A total of 477 patients were systematically evaluated based on predefined inclusion and exclusion criteria. Individuals aged 18 years or older who underwent diagnostic coronary angiography for suspected CAD and had complete clinical, laboratory, and imaging data at the time of hospital admission were included in the study. Patients with active infections, systemic inflammatory or autoimmune diseases, known malignancies, severe hepatic or renal dysfunction, recent major surgery or trauma within the past three months, use of immunosuppressive therapy, pregnancy, or incomplete data were excluded.

Based on angiographic and clinical findings, patients were categorized into three primary groups. The first group, the ACS group, included patients diagnosed according to the European Society of Cardiology guidelines for the management of ACSs. The second group, the CCS group, comprised patients classified according to the European Society of Cardiology guidelines for the diagnosis and management of CCSs. The third group, the control group, consisted of individuals presenting with similar symptoms but exhibiting normal epicardial coronary arteries on angiography. These symptoms primarily included non-specific chest discomfort or exertional complaints, which initially raised suspicion of either ACS or CCS but were ultimately not corroborated by angiographic or biochemical findings.

Demographic data, including age, sex, and body mass index (BMI), along with cardiovascular risk factors such as hypertension (HT), diabetes mellitus (DM), dyslipidemia (DL), active smoking, and a history of cerebrovascular disease, were recorded for each patient. Symptom onset times were also documented. Laboratory data were collected from samples taken within the first 24 h of hospital admission, typically prior to coronary angiography. Blood samples, including those for PCT, were obtained during this timeframe. Evaluated parameters included serum PCT, CRP, troponin-I, hemoglobin, total leukocyte and platelet counts, total cholesterol, low-density lipoprotein cholesterol (LDL-C), high-density lipoprotein cholesterol (HDL-C), triglycerides, and glomerular filtration rate (GFR). All laboratory analyses were conducted in the hospital’s central laboratory using standard biochemical analyzers and validated according to manufacturer protocols.

Serum PCT levels were measured using high-sensitivity electrochemiluminescence immunoassay (ECLIA) kits (Elecsys BRAHMS PCT, Roche Diagnostics GmbH, Mannheim, Germany) on a Cobas e601 analyzer. The analytical limit of detection (LOD) was 0.02 ng/mL, and the limit of quantitation (LOQ) was 0.06 ng/mL. The intra-assay and inter-assay coefficients of variation (CVs) were reported by the manufacturer as <6% and <8%, respectively, and verified in our laboratory. Blood samples were collected, centrifuged at 3,000 rpm for 10 min, and analyzed immediately or stored at –80 ^∘^C for batch analysis, ensuring optimal stability and reproducibility of PCT measurements.

Coronary angiography was performed on all patients via the femoral or radial approach using standard catheter techniques. The images obtained were retrospectively reviewed on the PACS system by two independent cardiologists with expertise in coronary anatomy assessment. During this review, the SYNTAX score was calculated for each patient, taking into account the number of lesions, anatomical localization, bifurcation structure, and presence of calcification. When inter-rater agreement reached ≥90%, the scores were averaged and included in the analysis; in cases of disagreement, a third senior cardiologist was consulted.

### Data analysis

#### Statistical analysis

Initially, the distribution characteristics of continuous variables were assessed using the Kolmogorov–Smirnov test for normality and Levene’s test for homogeneity of variances. Parametric tests, including one-way ANOVA, were conducted only on variables that satisfied both normality and homogeneity assumptions; otherwise, non-parametric alternatives, such as the Kruskal–Wallis test, were utilized. For variables exhibiting statistically significant differences, pairwise comparisons were executed using the post hoc Mann–Whitney *U* test. To mitigate the risk of Type I error due to multiple comparisons, a Bonferroni-adjusted significance threshold of *P* < 0.017 was employed, while the overall significance level was established at α ═ 0.05. Continuous variables were reported as mean ± standard deviation (SD) or median (interquartile range, IQR).

For categorical variables, frequencies and percentages were calculated, with comparisons among the three groups conducted using the Pearson chi-square test. In cases where more than 20% of expected cell frequencies were below 5, the Fisher exact test was utilized.

In alignment with the primary objective of the study, two distinct multivariate logistic regression analyses were performed to evaluate the independent effect of PCT levels in predicting the presence of ACS and CCS (CCS). The ACS model included patients diagnosed with both ST-elevation myocardial infarction (STEMI, *n* ═ 112) and non-ST-elevation myocardial infarction (NSTEMI, *n* ═ 78), while patients with unstable angina were excluded from the regression analysis. In these models, the dependent variables were defined as the presence of ACS and CCS, respectively. The independent variables encompassed age, sex, BMI, HT, DM, DL, smoking status, GFR, CRP, and PCT levels. Regression results were presented as odds ratios (OR) with 95% confidence intervals (CI) and *P* values. Multicollinearity among variables in the multivariate models was assessed using the variance inflation factor (VIF), with values <5 deemed acceptable; all included variables exhibited VIF values ranging from 1.2 to 2.8, indicating no significant collinearity. Linearity for continuous predictors was evaluated using the Box–Tidwell test, revealing no significant deviations. Influential observations were further scrutinized using standardized residuals and leverage values, with no cases exceeding commonly accepted thresholds.

To investigate the association between PCT levels and disease severity, correlation analyses were conducted. These analyses assessed the relationships between PCT and SYNTAX score, CRP, and troponin-I levels. Depending on the distribution characteristics of the variables, either Pearson’s correlation coefficient was employed (when parametric assumptions were satisfied), or Kendall’s Tau-b correlation coefficient was utilized (for non-parametric data). For the association between PCT and SYNTAX score in the CCS group, Pearson’s correlation was applied, as both variables satisfied parametric assumptions. Correlation strength was categorized as weak (*r* < 0.3), moderate (*r* ═ 0.3–0.6), or strong (*r* > 0.6), as defined in the relevant literature.

Additionally, patients in the ACS group were stratified into two subgroups based on serum PCT levels: PCT ≥0.25 ng/mL and PCT <0.25 ng/mL. These subgroups were compared regarding in-hospital mortality, one-year all-cause mortality, and the incidence of major adverse cardiovascular events (MACE). For categorical outcome variables, either the chi-square test or, when appropriate, the Fisher exact test was employed.

#### Software

All statistical analyses were conducted using IBM SPSS Statistics for Windows Version 21.0 (IBM Corp., Armonk, NY, USA) and MedCalc Statistical Software Version 19.2.6 (MedCalc Software Ltd., Ostend, Belgium). A *P* value of less than 0.05 was deemed statistically significant. Additionally, a post hoc power analysis was performed using G*Power software. Based on the regression model (Nagelkerke R^2^ ═ 0.27), the calculated power was 93.6% for detecting the predictor effect size at an alpha level of 0.05.

### Ethical approval

Ethical approval for the study was obtained from the Non-Interventional Clinical Research Ethics Committee of Mersin University Faculty of Medicine, with decision number 2020/346 dated April 29, 2020.

### Declaration of Helsinki

The research process was conducted entirely in accordance with the ethical principles of the Declaration of Helsinki, published in 2013 by the World Medical Association, and all scientific procedures were carried out within this framework.

### Informed written consent

Written informed consent was obtained from all individuals included in the study as part of standard clinical procedures at the time of hospitalization. This consent form explicitly covered both the treatment process and the analysis of data to be used in this study.

## Results

A total of 477 patients participated in this study, classified into three groups based on angiographic findings: the ACS group (*n* ═ 190), the CCS group (*n* ═ 202), and the control group (*n* ═ 85). We compared demographic characteristics, clinical risk factors, laboratory parameters, and angiographic scores across these groups. The normality of distribution for continuous variables was assessed, and appropriate statistical tests were employed. Furthermore, we evaluated the relationship between PCT levels and clinical markers using correlation analysis and analyzed the prognostic value of PCT within subgroups of the ACS cohort.

The results are organized under the subheadings of demographic findings, laboratory parameters, correlation analyses, and multivariate logistic regression results.

Table 1 shows that the mean age was highest in the ACS group (64.2 ± 10.8 years) and lowest in the control group (57.1 ± 13.3 years), with a statistically significant difference (*P* < 0.001). The proportion of male patients was significantly greater in both the ACS (70.5%) and CCS (68.3%) groups compared to the control group (42.4%) (*P* < 0.001). Left ventricular ejection fraction (LVEF) was lower in the ACS group [median 50.0 (45.0–55.0)] compared to both the CCS and control groups [median 60.0 (55.0–60.0) and 60.0 (55.0–62.5), respectively], with a significant difference (*P* < 0.001). The white blood cell (WBC) count was highest in the ACS group [median 9.1 (7.6–11.4)] and lowest in the control group [median 7.1 (5.5–8.0)] (*P* < 0.001). Platelet counts varied significantly among the groups, with higher values in the CCS group [median 249.0 (215.8–308.0)] (*P* ═ 0.002). The GFR was significantly higher in the control group [median 97.5 (89.7–107.5)] compared to the ACS and CCS groups (*P* ═ 0.001). HDL cholesterol levels were significantly lower in the ACS group [median 39.6 (34.0–46.2)] compared to controls [median 47.0 (39.8–54.0)] (*P* < 0.001). Triglyceride levels were highest in the CCS group [median 151.0 (99.8–211.0)] and differed significantly across groups (*P* ═ 0.002). DL occurred significantly more frequently in the CCS group (90.1%) compared to the control group (*P* < 0.001); however, differences between the ACS and CCS groups did not remain statistically significant after Bonferroni correction (*P* ═ 0.034 for ACS vs control, *P* ═ 0.058 for ACS vs CCS). PCT levels were significantly elevated in both the ACS and CCS groups [median 0.320 (0.18–0.60) and 0.320 (0.17–0.52), respectively] compared to controls [median 0.160 (0.05–0.24)] (*P* < 0.001). Similarly, CRP levels were significantly higher in the ACS group [median 3.94 (1.65–8.70)] than in the CCS and control groups (*P* ═ 0.001). Smoking prevalence was greater in the ACS group (32.6%) compared to the CCS (19.8%) and control (17.6%) groups (*P* ═ 0.003). DM was more common in the CCS group (44.5%) than in the control group (*P* < 0.001) and the ACS group (*P* ═ 0.003); however, the difference between the ACS and control groups (*P* ═ 0.027) did not achieve statistical significance after Bonferroni correction. Finally, the SYNTAX score was significantly higher in the ACS group (18.5 ± 9.4) compared to the CCS group (12.2 ± 8.2) (*P* < 0.001).

**Table 1:** Comparison of demographic, clinical, and laboratory parameters among ACS, CCS, and control groups (*n* ═ 477)

**Variable**	**ACS group (*n* ═ 190)**	**CCS group (*n* ═ 202)**	**Control group (*n* ═ 85)**	***P* value**
Age (years)	64.2 ± 10.8	62.4 ± 9.3	57.1 ± 13.3	**<0.001**
Male sex, *n*(%)	134 (70.5%)	138 (68.3%)	36 (42.4%)	**<0.001**
Systolic blood pressure (mmHg)	131.8 ± 20.4	133.7 ± 17.3	128.0 ± 15.2	0.062
Diastolic blood pressure (mmHg)	76.0 (70.0–81.5)	78.0 (72.0–84.3)	77.0 (71.0–89.0)	0.143
LVEF (%)	50.0 (45.0–55.0)	60.0 (55.0–60.0)	60.0 (55.0–62.5)	**<0.001**
WBC (×10^3^/mL)	9.1 (7.6–11.4)	8.0 (6.7–9.4)	7.1 (5.5–8.0)	**<0.001**
Platelet count (×10^3^/mL)	240.0 (188.3–275.0)	249.0 (215.8–308.0)	246.0 (202.5–295.5)	**0.002**
Hemoglobin (g/dL)	13.45 (11.9–15.05)	13.8 (12.2–14.7)	13.3 (11.6–14.5)	0.128
GFR (mL/min/1.73 m^2^)	92.4 (75.1–102.0)	91.9 (73.3–101.0)	97.5 (89.7–107.5)	**0.001**
Total cholesterol (mg/dL)	185.8 ± 47.8	189.9 ± 52.5	200.0 ± 44.9	0.088
LDL cholesterol (mg/dL)	117.4 ± 40.4	114.9 ± 46.2	121.3 ± 36.9	0.231
HDL cholesterol (mg/dL)	39.6 (34.0–46.2)	40.6 (34.8–46.7)	47.0 (39.8–54.0)	**<0.001**
Triglycerides (mg/dL)	123.0 (80.0–177.3)	151.0 (99.8–211.0)	130.0 (94.0–168.0)	**0.002**
PCT (ng/mL)	0.320 (0.18–0.60)	0.320 (0.17–0.52)	0.160 (0.05–0.24)	**<0.001**
CRP (mg/L)	3.94 (1.65–8.70)	2.74 (1.25–58.85)	2.40 (1.0–5.0)	**0.001**
BMI (kg/m^2^)	27.1 (25.4–31.1)	27.5 (24.9–31.2)	26.8 (24.6–30.3)	0.197
Smoking, *n*(%)	62 (32.6%)	40 (19.8%)	15 (17.6%)	**0.003**
Hypertension, *n*(%)	103 (54.2%)	128 (63.4%)	42 (49.4%)	0.057
History of cerebrovascular disease, *n*(%)	4 (2.1%)	5 (2.5%)	2 (2.4%)	0.982
Diabetes mellitus, *n*(%)	57 (30.0%)	90 (44.5%)	18 (21.2%)	**<0.001**
Dyslipidemia, *n*(%)	159 (83.7%)	182 (90.1%)	61 (71.7%)	**<0.001**
SYNTAX score	18.5 ± 9.4	12.2 ± 8.2	–	**<0.001**

Statistical tests applied include one-way ANOVA for normally distributed continuous variables, Kruskal-Wallis test for non-normally distributed variables, and chi-square test or Fisher’s exact test for categorical data. Post hoc analyses were performed using Mann–Whitney *U* test where applicable. In the table, statistically significant *P* values (*P* < 0.05) are marked in **bold**. The *P* value indicates the level of statistical significance. Abbreviations: LVEF: Left ventricular ejection fraction; WBC: White blood cell count; GFR: Glomerular filtration rate; PCT: Procalcitonin; CRP: C-reactive protein; BMI: Body mass index; LDL: Low-density lipoprotein; HDL: High-density lipoprotein; ACS: Acute coronary syndrome; CCS: Chronic coronary syndrome.

According to the results presented in Table 2, post-hoc analysis revealed a statistically significant difference in age between the CCS and control groups (*P* < 0.001) and between the ACS and control groups (*P* < 0.001). Post-hoc analyses were performed among the three groups: ACS (*n* ═ 190), CCS (*n* ═ 202), and control (*n* ═ 85) to identify the sources of these significant differences.

**Table 2:** Post hoc comparison of statistically significant variables among CCS (*n* ═ 202), ACS (*n* ═ 190), and control (*n* ═ 85) groups

**Variable**	**CCS vs control (*P*)**	**ACS vs control (*P*)**	**CCS vs ACS (*P*)**
Age	**<0.001**	**<0.001**	0.045
Male sex	**<0.001**	**<0.001**	0.368
LVEF (%)	0.212	**<0.001**	**<0.001**
WBC (×10^3^/mL)	**<0.001**	**<0.001**	**<0.001**
Platelet count (×10^3^/mL)	0.103	0.076	**<0.001**
GFR (mL/min/1.73 m^2^)	**<0.001**	**0.001**	0.089
HDL cholesterol (mg/dL)	**<0.001**	**<0.001**	0.064
Triglycerides (mg/dL)	0.098	0.116	**<0.001**
PCT (ng/mL)	**<0.001**	**<0.001**	0.029
CRP (mg/L)	0.054	**0.001**	**0.002**
Smoking	0.061	**0.001**	**0.004**
Diabetes mellitus	**<0.001**	0.027	**0.003**
Dyslipidemia	**<0.001**	0.034	0.058

Statistical tests applied include one-way ANOVA for normally distributed continuous variables and chi-square test for categorical variables. Post hoc comparisons were performed using the Bonferroni-adjusted Mann–Whitney *U* test or pairwise chi-square test when appropriate. A Bonferroni-adjusted significance level of *P* < 0.017 was applied for multiple pairwise comparisons. In the table, statistical values that are significant are marked in **bold**. The *P* value indicates the level of statistical significance, with values less than 0.017 considered statistically significant after Bonferroni correction. Abbreviations: LVEF: Left ventricular ejection fraction; WBC: White blood cell count; GFR: Glomerular filtration rate; PCT: Procalcitonin; CRP: C-reactive protein; HDL: High-density lipoprotein; CCS: Chronic coronary syndrome; ACS: Acute coronary syndrome.

The proportion of male patients was significantly higher in both the CCS vs control (*P* < 0.001) and ACS vs control (*P* < 0.001) comparisons. LVEF was significantly lower in the ACS group compared to both the control and CCS groups (*P* < 0.001 for both comparisons). WBC count differed significantly among all pairwise comparisons (CCS vs control, ACS vs control, CCS vs ACS; *P* < 0.001 for each). Platelet count was significantly higher in the CCS group compared to the ACS group (*P* < 0.001).

GFR was significantly higher in the control group compared to both the CCS (*P* < 0.001) and ACS (*P* ═ 0.001) groups. High-density lipoprotein (HDL) cholesterol levels were significantly elevated in the control group relative to both CCS (*P* < 0.001) and ACS (*P* < 0.001). Triglyceride levels were significantly higher in CCS compared to ACS (*P* < 0.001). PCT levels were significantly elevated in both the CCS and ACS groups when compared to the control group (*P* < 0.001 for both comparisons).

CRP levels were significantly higher in the ACS group [median 3.94 (1.65–8.70)] compared to the control group (*P* ═ 0.001) and also significantly higher than in the CCS group (*P* ═ 0.002; Bonferroni threshold *P* < 0.017). Notably, the upper quartile boundary for CRP in the CCS group was 58.85 mg/L, indicating a markedly right-skewed distribution with a heavy upper tail rather than isolated outliers. All results were corroborated by original laboratory records. Given that nonparametric statistical tests were utilized for group comparisons, these outliers did not compromise the analyses’ robustness.

In the post-hoc comparisons, a Bonferroni-adjusted significance threshold of *P* < 0.017 was employed due to multiple pairwise tests. Some findings with *P* values marginally exceeding this threshold (e.g., *P* ═ 0.02) were not deemed statistically significant under this correction, though they may suggest a trend. Caution is warranted in interpreting these borderline results to avoid overestimating their clinical or statistical relevance.

Smoking prevalence was significantly higher in the ACS group compared to both the control (*P* ═ 0.001) and CCS (*P* ═ 0.004) groups. Additionally, DM was more prevalent in the CCS group than in both the control (*P* < 0.001) and ACS groups (*P* ═ 0.003). DL was also significantly more common in the CCS group compared to the control group (*P* < 0.001).

The data presented in Table 3 indicate that advancing age is significantly correlated with an increased likelihood of CCS (OR: 1.047, 95% CI: 1.01–1.08, *P* ═ 0.008). This analysis adjusted for several covariates, including age, sex, BMI, HT, DM, DL, smoking status, GFR, CRP, and PCT. Male sex emerged as a significant predictor, with men being over four times more likely to have CCS compared to women (OR: 4.284, 95% CI: 2.10–8.76, *P* < 0.001). DL was also independently associated with CCS, increasing the odds more than threefold (OR: 3.471, 95% CI: 1.42–8.47, *P* ═ 0.006). Notably, PCT levels demonstrated a statistically significant and independent association with CCS (OR: 2.81, 95% CI: 1.43–5.54, *P* < 0.001), indicating that elevated PCT levels are linked to nearly a threefold increase in the likelihood of CCS. Other variables, including BMI, DM, HT, smoking status, CRP, and GFR, did not achieve statistical significance in this model.

**Table 3:** Multivariate logistic regression analysis for predictors of chronic coronary syndrome (CCS) (*n* ═ 202)

**Variable**	**OR**	**95% CI**	***P* value**
Age (years)	1.047	1.01–1.08	**0.008**
Male sex	4.284	2.10–8.76	**<0.001**
BMI	1.017	0.95–1.09	0.326
DM	2.128	0.99–4.60	0.052
HT	1.277	0.63–2.57	0.492
Smoking	1.547	0.66–3.60	0.313
CRP	0.993	0.90–1.09	0.891
Dyslipidemia	3.471	1.42–8.47	**0.006**
GFR	0.982	0.96–1.00	0.078
PCT*	2.81	1.43–5.54	**<0.001**

Statistical tests applied include multivariate logistic regression analysis to determine independent predictors of chronic coronary syndrome (CCS). ^*^Due to the extremely high odds ratios observed when using raw PCT values in ng/mL, a log_10_ transformation of procalcitonin was applied to normalize the distribution and provide biologically plausible effect sizes. This transformation yielded a more interpretable odds ratio indicating that each 10-fold increase in PCT level was associated with a nearly 3-fold increase in the likelihood of CCS. In the table, statistical values that are significant are marked in **bold**. The *P* value indicates the level of statistical significance, where values less than 0.05 are considered significant. Abbreviations: OR: Odds ratio; CI: Confidence interval; BMI: Body mass index; DM: Diabetes mellitus; HT: Hypertension; CRP: C-reactive protein; GFR: Glomerular filtration rate; PCT: Procalcitonin.

The results presented in Table 4 reveal several statistically significant independent predictors of ACS. The multivariate logistic regression model for ACS was adjusted for a consistent set of variables: age, sex, BMI, HT, DM, DL, smoking status, GFR, CRP, and PCT levels.

**Table 4:** Multivariate logistic regression analysis for predictors of acute coronary syndrome (ACS) (*n* ═ 190)

**Variable**	**OR**	**95% CI**	***P* value**
Age (years)	1.108	1.06–1.15	**<0.001**
Male sex	7.498	3.10–18.16	**<0.001**
BMI	1.085	0.99–1.18	0.078
DM	3.207	1.29–7.99	**0.012**
HT	0.543	0.24–1.22	0.137
Smoking	4.124	1.58–10.73	**0.004**
CRP	1.112	1.02–1.21	**0.011**
Dyslipidemia	3.444	1.34–8.86	**0.010**
GFR	0.990	0.97–1.01	0.273
PCT*	4.30	2.00–9.20	**<0.001**

Statistical tests applied include multivariate logistic regression analysis to determine independent predictors of acute coronary syndrome (ACS). ^*^Due to the extremely high odds ratios observed when using raw PCT values in ng/mL, a log_10_ transformation of procalcitonin was applied to normalize the distribution and provide biologically plausible effect sizes. This transformation yielded a more interpretable odds ratio indicating that each 10-fold increase in PCT level was associated with a approximately 4-fold increase in the likelihood of ACS. In the table, statistical values that are significant are marked in **bold**. The *P* value indicates the level of statistical significance, where values less than 0.05 are considered significant. Abbreviations: OR: Odds ratio; CI: Confidence interval; BMI: Body mass index; DM: Diabetes mellitus; HT: Hypertension; CRP: C-reactive protein; GFR: Glomerular filtration rate; PCT: Procalcitonin.

**Table 5:** ROC curve analysis for procalcitonin (PCT) in predicting coronary syndromes (*n* ═ 477)

**Comparison group**	**Sensitivity (%)**	**Specificity (%)**	**AUC**	***P* value**	**Cut-off (ng/mL)**
CCS vs control group	89.4	54.0	0.763	**<0.001**	0.30
ACS vs control group	82.4	65.3	0.791	**<0.001**	0.25

Statistical tests applied include receiver operating characteristic (ROC) curve analysis to evaluate the diagnostic performance of procalcitonin (PCT) in differentiating coronary syndromes. Cut-off values represent the thresholds above which procalcitonin levels were considered elevated for diagnostic classification in ROC analysis. In the table, values that are statistically significant are marked in **bold**. The *P* value indicates the level of statistical significance, where values less than 0.05 are considered significant. Abbreviations: AUC: Area under the curve; ACS: Acute coronary syndrome; CCS: Chronic coronary syndrome; PCT: Procalcitonin.

**Table 6:** In-hospital and one-year adverse cardiovascular outcomes in ACS patients stratified by PCT levels

**Outcome**	**PCT ≥0.25 ng/mL (*n*, %)**	**PCT <0.25 ng/mL (*n*, %)**	***P* value**
In-hospital mortality	2 (1.6%)	1 (1.6%)	0.994
1-year all-cause mortality	9 (7.0%)	5 (8.2%)	0.743
1-year major adverse cardiovascular events	12 (9.3%)	7 (11.5%)	0.624

Statistical tests applied include Pearson’s chi-square test and Fisher’s exact test for categorical variables. In the table, statistical values that are significant are marked in **bold**. The *P* value indicates the level of statistical significance, where values less than 0.05 are considered statistically significant. Abbreviations: ACS: Acute coronary syndromes; PCT: Procalcitonin; MACE: Major adverse cardiovascular events.

Increasing age was strongly correlated with the risk of ACS, with an odds ratio (OR) of 1.108 (95% CI: 1.06–1.15, *P* < 0.001). Male participants demonstrated a notably elevated OR, indicating that men are over seven times more likely to present with ACS compared to women (OR: 7.498, 95% CI: 3.10–18.16, *P* < 0.001). Additionally, DM was significantly associated with ACS (OR: 3.207, 95% CI: 1.29–7.99, *P* ═ 0.012).

Smoking was found to increase the risk of ACS by more than fourfold (OR: 4.124, 95% CI: 1.58–10.73, *P* ═ 0.004), while DL was linked to a threefold increase in risk (OR: 3.444, 95% CI: 1.34–8.86, *P* ═ 0.010). Elevated levels of CRP were associated with a higher likelihood of developing ACS (OR: 1.112, 95% CI: 1.02–1.21, *P* ═ 0.011). Importantly, PCT emerged as a robust and independent predictor of ACS, with an OR of 4.30 (95% CI: 2.00–9.20, *P* < 0.001), indicating that increasing PCT levels correspond to a more than fourfold rise in the risk of ACS.

The data presented in Table 5 demonstrate that PCT exhibits strong discriminatory ability in differentiating CCS and ACS from control patients. In the CCS group, PCT levels showed a sensitivity of 89.4% and a specificity of 54.0%, with an AUC of 0.763 and a statistically significant *P* value of <0.001. For the ACS group, sensitivity was slightly lower at 82.4%, while specificity increased to 65.3%. The AUC for this group was 0.791, with a *P* value also <0.001. The optimal cut-off values for PCT were determined to be 0.30 ng/mL for CCS and 0.25 ng/mL for ACS. Consequently, in subgroup analyses, patients with PCT levels ≥0.25 ng/mL were categorized as having elevated PCT. These findings suggest that PCT may serve as a moderately accurate biomarker for distinguishing both chronic and ACSs from patients without angiographic evidence of CAD.

Table 6 presents the in-hospital and one-year clinical outcomes of patients diagnosed with ACS, stratified by PCT levels. Patients with PCT levels ≥0.25 ng/mL exhibited an in-hospital mortality rate of 1.6%, comparable to the rate for those with PCT <0.25 ng/mL. The one-year all-cause mortality was 7.0% in the high PCT group and 8.2% in the low PCT group. Furthermore, the one-year MACE rate was 9.3% in the elevated PCT group and 11.5% in the lower PCT group. No statistically significant differences were observed between the two groups across any of the outcome measures.

## Discussion

CAD, both in its acute and chronic forms, remains one of the leading causes of morbidity and mortality globally. It is well established that inflammation plays a central role in the development of cardiovascular events, with the degree and duration of the inflammatory response directly influencing the clinical presentation and prognosis [12, 13]. Consequently, investigating the diagnostic and prognostic significance of inflammatory markers is a critical area of research that has the potential to enhance clinical management. This study aimed to compare PCT levels in patients with acute and CCSs, evaluate the relationship between these levels and clinical as well as angiographic parameters, and determine their predictive value.

In this study, we analyzed 477 patients, categorizing them into three groups: ACS, CCS, and a control group. Our findings indicate that serum PCT levels were significantly elevated in both ACS and CCS patients; however, no significant difference was observed between the two disease groups. Furthermore, PCT levels in the CCS group exhibited a weak yet statistically significant correlation with the SYNTAX score. In contrast, no significant relationship was identified between PCT levels and the SYNTAX score, CRP, or troponin-I in the ACS group.

PCT is a well-established biomarker frequently elevated in bacterial infections. However, elevated PCT levels have also been reported in systemic inflammatory processes unrelated to infection [10]. The pathophysiology underlying non-infectious PCT elevation has been elucidated, particularly through mechanisms such as hypoxic stress, endothelial damage, cytokine release, and neural stress [14]. In cardiac diseases, these mechanisms are activated during episodes of myocardial ischemia and reperfusion. Pro-inflammatory cytokines, including IL-6 and tumor necrosis factor-alpha (TNF-α), directly enhance PCT expression, a process that can occur independently of infection [15, 16].

From this perspective, PCT may indicate not only infection but also cardiovascular inflammation. For instance, a 2021 study by Sharma et al. [17] found significantly elevated PCT levels in patients with ST-elevation myocardial infarction, particularly in cases complicated by cardiogenic shock, and this increase was significantly associated with prognosis. Similarly, a 2022 prospective study by Pavasini et al. [18] indicated that while PCT was not as robust a prognostic marker as CRP or IL-6 in ACS patients, its levels were still significantly higher in high-risk individuals. Consistent with these findings, our study also demonstrated significantly elevated PCT levels in ACS patients compared to controls; however, the lack of correlation with the SYNTAX score, troponin-I, or CRP suggests that in acute coronary events, PCT may reflect a systemic inflammatory response rather than disease severity.

Conversely, some studies have reported more pronounced associations. A 2022 study by Hu et al. identified a link between high PCT levels and 30-day mortality in ACS patients. Similarly, a 2019 study by Clementi et al. reported a significant association between elevated PCT levels and in-hospital mortality among patients undergoing cardiac surgery [6, 19]. Supporting these findings, a 2024 prospective observational study by Hassan et al. demonstrated that plasma PCT levels in patients with acute ST-elevation myocardial infarction were significantly associated with the occurrence of MACE, and that elevated levels could predict poor prognosis in the early phase [20]. In these studies, a PCT threshold of ≥0.25 ng/mL was commonly utilized, with levels above this threshold emphasized for their clinical significance.

In our study, the ACS group was divided into two subgroups based on a PCT cut-off of 0.25 ng/mL. However, no significant differences were observed between these subgroups regarding mortality and MACE. It is important to note that the number of outcome events in our cohort—specifically, in-hospital and one-year mortality—was relatively low, with only 3 and 14 cases, respectively. Due to this limited number of in-hospital and one-year deaths, we did not apply multivariable regression models for mortality or MACE. Instead, we performed only categorical comparisons using chi-square and Fisher’s exact test, as presented in Table 6. This methodological approach restricts our ability to draw definitive prognostic conclusions.

According to established methodological standards, logistic regression analysis typically requires approximately 10 outcome events per predictor variable to ensure statistical validity. Our cohort’s limited number of outcome events did not satisfy this criterion, prompting us to refrain from outcome modeling to prevent the generation of misleading or unstable estimates. This limitation suggests that the prognostic value of PCT in ACS may be influenced by various factors, including disease severity, timing of presentation, comorbid conditions, hemodynamic instability, concomitant infections, and systemic inflammation. Furthermore, the overall low event rate in our cohort may have reduced the statistical power to identify significant differences in clinical outcomes based on PCT levels. Additionally, as indicated by some studies, the prognostic significance of PCT may become more pronounced in ACS cases complicated by sepsis or concurrent infections [21, 22].

The findings from the CCS group represent a less frequently explored yet increasingly significant area of research. Systemic inflammation has been shown to persist at low levels in patients with stable angina pectoris, contributing to the progression of atherosclerotic plaques [23]. A study by Alavi et al. [24] employing advanced positron emission tomography (PET) imaging techniques demonstrated inflammatory cell infiltration, including T cells and macrophages, even in stable atherosclerotic lesions; these infiltrates correlated with systemic inflammatory activity. The modest but statistically significant correlation identified between PCT levels and the SYNTAX score in our CCS group may reflect systemic inflammatory activity associated with anatomical disease burden. Conversely, this correlation was not observed in the ACS group, potentially due to the dynamic and time-sensitive nature of inflammation during acute events, which may not directly correlate with anatomical burden as measured by the SYNTAX score. Notably, in multivariate analyses, both DL and elevated PCT levels were significantly associated with disease presence in the CCS group. Identifying PCT as an independent predictor in CCS suggests that this biomarker may reflect the inflammatory burden in both acute and CCSs. Each increase in PCT level corresponded to a marked increase in disease probability, aligning with limited literature highlighting the role of systemic inflammation in CCS.

The association between PCT and the SYNTAX score has been examined in only a few studies. A 2017 study by Ertem et al. [25] revealed a significant positive correlation between serum PCT levels and the SYNTAX score in patients with ACS, indicating that PCT may reflect the severity and complexity of CAD. This finding supports our results in the CCS group. The observation that each incremental rise in PCT correlates with a moderate increase in disease likelihood warrants further investigation. In the ACS group analysis, variables, such as age, male sex, DM, smoking, DL, and CRP, were significantly associated with disease presence, with PCT levels emerging as particularly impactful. A serum PCT concentration of ≥0.25 ng/mL was associated with the presence of ACS in this cohort. ROC analysis supported these findings, identifying 0.25 ng/mL and 0.30 ng/mL as optimal cutoff values for ACS and CCS, respectively, with AUC values of 0.791 and 0.763. These data suggest that PCT may serve not only as a marker of inflammatory burden but also as a supportive biomarker in diagnosing CCS and ACS. However, this relationship was not observed in the ACS group, potentially indicating that PCT levels in the acute phase reflect the temporal stage of systemic inflammation rather than disease burden.

CRP is one of the most commonly used inflammatory markers in CVD contexts. However, since CRP is of hepatic origin and rises later, it may be limited in identifying and stratifying early acute inflammation. In contrast, PCT rises within a few hours and has a half-life of 20–24 h [26], making it valuable in the early inflammatory response. However, its higher cost compared to CRP restricts its routine use. The absence of a significant correlation between PCT and CRP in the ACS group of our study supports the notion that these two markers reflect different biological windows.

Another noteworthy finding is the lack of correlation between PCT and troponin-I levels. While troponin directly indicates myocardial cell injury, PCT reflects systemic inflammation. The dissociation between these markers suggests that PCT is more sensitive to inflammatory responses than to myocardial necrosis [27]. Our study found no significant correlation between PCT and troponin-I levels, reinforcing the idea that PCT is more closely linked to inflammation-based processes than to myocardial damage.

A strength of our study is the evaluation of PCT levels in a patient population where infectious causes were excluded, allowing for a clearer assessment of PCT’s role in non-infectious cardiac events. However, it should be noted that the control group, although free of angiographically evident CAD, included individuals with common cardiovascular risk factors such as DL and HT. While these patients did not have overt CAD, they may still represent a population with potential subclinical atherosclerosis. This characteristic should be considered when interpreting intergroup differences, as it may have influenced the observed levels of inflammatory biomarkers such as PCT. Additionally, the relatively large sample size and the use of multivariate analyses to control for confounding factors enhance the scientific validity of our study.

This research demonstrates that PCT levels are significantly elevated in both acute and CCSs and are modestly associated with disease extent in CCS. Compared to other biomarkers such as CRP and troponin, PCT reflects distinct biological pathways and offers diagnostic or differential value. ROC analysis results indicated moderate diagnostic power, highlighting PCT’s potential utility as a diagnostic tool, particularly when rapid decision-making is required and other inflammatory markers are unavailable or delayed. The cutoff values were determined to be 0.25 ng/mL for ACS and 0.30 ng/mL for CCS, with patients having PCT ≥0.25 ng/mL classified into the elevated ACS subgroup, consistent with subgroup analyses and Table 5. These findings suggest that PCT may play a supportive role in diagnosis.

However, the role of PCT in predicting cardiovascular prognosis remains controversial, and it is not a standalone determinant in clinical decision-making. Nonetheless, in multivariate logistic regression analysis of the ACS group, age, male sex, diabetes, smoking, DL, CRP, and PCT levels were significantly associated with disease presence. This suggests that PCT may be useful not only for reflecting inflammation but also for identifying high-risk patient profiles. PCT emerged as a significant predictor of ACS, with an adjusted OR of 4.30 (95% CI: 2.00–9.20), indicating a meaningful increase in risk with rising PCT levels. Therefore, PCT should be utilized within a multidisciplinary framework alongside other inflammatory and cardiac biomarkers.

### Limitations of the study

The findings of this study should be interpreted with caution due to several methodological and structural limitations. Firstly, the retrospective, single-center design represents a significant constraint. This observational design precludes the establishment of causal relationships between elevated PCT levels and clinical outcomes or disease presence; thus, the findings should be regarded as associative rather than causal. As data were derived from archived records, prospective control over clinical variables was not achievable, and potential confounding factors may have been overlooked. Furthermore, the study population was sourced from a single hospital, which may limit the demographic and clinical diversity of the sample, thereby restricting the generalizability of the findings.

Secondly, PCT levels were assessed only at the time of admission, with no monitoring of temporal changes. The kinetic variation of inflammatory markers over time, particularly in dynamic scenarios such as ACS, could yield valuable clinical insights. Serial monitoring of PCT levels could have elucidated its prognostic role and its relationship with clinical progression more clearly.

Thirdly, while in-hospital and one-year mortality rates and MACE were reported, these outcomes were analyzed solely using univariate categorical tests, and the low event count rendered the analyses underpowered. Consequently, this study cannot draw definitive conclusions regarding the prognostic significance of PCT. The evaluation of PCT was limited to its admission values, preventing an analysis of its long-term prognostic implications. This limitation hindered a comprehensive assessment of PCT’s potential contribution to clinical follow-up and risk stratification.

Fourthly, certain systemic conditions that may elevate PCT independently of infection (e.g., trauma, autoimmune diseases, or subclinical infections) may not have been entirely excluded. Although patients with overt infections were carefully excluded, it is conceivable that subclinical or undiagnosed inflammatory conditions could have influenced PCT levels, representing a potential confounding factor. Despite meticulous review of clinical records and laboratory findings, achieving complete specificity for a parameter as sensitive as PCT in response to various biological stimuli is challenging in a retrospective study design.

Fifthly, the study evaluated only a single inflammatory biomarker (PCT) without comparative analysis against other significant biomarkers (e.g., IL-6, pro-BNP, presepsin, or hs-CRP). A comprehensive evaluation of multiple biomarkers could have provided a more nuanced understanding of the inflammatory process and improved diagnostic accuracy. Additionally, the ROC-derived cut-off values reported in this study should be viewed as exploratory, as no internal (e.g., bootstrapping or cross-validation) or external validation was performed, nor was there a formal calibration assessment (e.g., Hosmer–Lemeshow test). Therefore, these cut-offs may be overly optimistic and necessitate confirmation in independent cohorts.

Lastly, the study did not thoroughly control for patients’ medical treatments (e.g., statin use, ACE inhibitors, and antibiotics), which are known to influence inflammatory marker levels. Failure to account for such variables may have biased the results by affecting the measured values of sensitive parameters like PCT.

Despite these limitations, this study is one of the few exploring PCT in both acute and CCS patients, contributing valuable insights to the existing literature. To derive more robust conclusions, prospective, multicenter studies involving larger patient populations are essential.

## Conclusion

This study demonstrated that PCT levels were significantly elevated in both ACS and CCS patients compared to controls with angiographically normal epicardial coronary arteries. In both patient groups, elevated PCT levels indicated the presence of systemic inflammation. However, in the ACS group, PCT levels did not demonstrate a significant relationship with disease extent as measured by the SYNTAX score, myocardial injury indicated by troponin-I levels, or CRP levels. In contrast, a weak but positive correlation was observed between PCT levels and the SYNTAX score in the CCS group. Multivariate logistic regression analyses identified PCT as an independent predictor for both types of coronary syndromes. These findings suggest that PCT may serve as a biomarker reflecting not only infectious conditions but also inflammation associated with atherosclerotic processes.

Nonetheless, the prognostic value of PCT appears limited, indicating that it may not be sufficient as a standalone parameter in clinical decision-making. In ACS patients, PCT levels exceeding the threshold of ≥0.25 ng/mL did not significantly predict prognosis. Consequently, PCT should be interpreted within an integrated framework that includes other biomarkers and anatomical scoring systems. In this context, the routine clinical use of PCT as a standalone biomarker in CAD evaluation is not currently supported, and its implementation should be considered only as part of a broader diagnostic algorithm. Given its potential to reflect inflammatory responses, PCT may serve as a useful biochemical indicator for assessing inflammatory activity, particularly in relatively asymptomatic conditions such as CCS. However, this assertion necessitates further validation through prospective, multicenter studies with larger sample sizes.

## Supplemental data

### Key points

#### What is known about the topic?

Procalcitonin (PCT) is a well-established biomarker commonly used to detect bacterial infections and assess systemic inflammation. Recent studies have suggested that PCT may also increase in non-infectious inflammatory conditions such as acute coronary syndromes (ACS), potentially reflecting the intensity of systemic inflammation. However, the data on its diagnostic and prognostic utility in both acute and chronic coronary syndromes (CCS) are limited and often conflicting. Moreover, its association with anatomical disease severity, such as quantified by SYNTAX score, has not been fully elucidated.

#### What does this study add?

This study provides comparative clinical evidence that serum PCT levels are significantly elevated in both ACS and CCS patients compared to controls, supporting its role as a non-infectious inflammatory marker in atherosclerotic disease. Importantly, it identifies PCT as an independent predictor for the presence of both ACS and CCS through multivariate logistic regression analysis. Furthermore, a weak but significant correlation between PCT and SYNTAX score was observed in CCS patients, suggesting that PCT may reflect chronic atherosclerotic burden. Despite these associations, PCT was not predictive of prognosis (mortality or MACE) in ACS patients, highlighting its limited role as a standalone prognostic marker.

## Data Availability

All datasets generated during this study are available from the corresponding author upon reasonable request. In addition, catheterization laboratory logs and hospital information system summaries used to confirm angiography volumes during the recruitment period can also be provided upon request.
